# Translation, cross-cultural adaptation and validation of SinoNasal Outcome Test (SNOT) - 22 to Brazilian Portuguese

**DOI:** 10.1590/S1808-86942011000500021

**Published:** 2015-10-22

**Authors:** Eduardo Macoto Kosugi, Vitor Guo Chen, Viviane Maria Guerreiro da Fonseca, Milena Martins Pellogia Cursino, José Arruda Mendes Neto, Luís Carlos Gregório

**Affiliations:** 1 MSc in Sciences, Chief Preceptor of the ENT Residency Program - UNIFESP-EPM (Paulista School of Medicine - Federal University of São Paulo); 2 MD - PUC-Sorocaba; 3rd-Year ENT Resident - UNIFESP-EPM; 3 MD - UNIFESP-EPM; 3rd-Year ENT Resident - UNIFESP-EPM; 4 6th-Year Medical Student - UNIFESP-EPM; 5 MD. ENT - UNIFESP-EPM; MSc Student - UNIFESP-EPM; 6 Associate Professor of Rhino-Laryngology UNIFESP-EPM, Vice-Chief - Department of Otorhinolaryngology and Head and Neck Surgery - UNIFESP-EPM

**Keywords:** nasal polyps, natural orifice endoscopic surgery, quality of life, questionnaires, sinusitis

## Abstract

**Abstract:**

Quality of life questionnaires have been increasingly used in clinical trials to help establish the impact of medical intervention or to assess the outcome of health care services. Among disease-specific outcome measures, SNOT-22 was considered the most suitable tool for assessing chronic rhinosinusitis and patients with nasal polyps.

**Aims:**

To perform translation, cross-cultural adaptation and validation of the SNOT-22 to Brazilian Portuguese.

**Methods:**

Prospective study involving eighty-nine patients with chronic rhinosinusitis or nasal polyps submitted to functional endoscopic sinus surgery, who answered the questionnaire before and after surgery. Furthermore, 113 volunteers without sinonasal disease also answered the questionnaire. Internal consistency, test-retest reliability, measure validity, responsiveness and clinical interpretability were assessed.

**Results:**

Mean preoperative, postoperative and no sinonasal disease scores were 62.39, 23.09 and 11.42, respectively (*p*<0.0001); showing validity and responsiveness. Internal consistency was high (Cronbach's alpha = 0.9276). Reliability was sufficiently good, considering inter-interviewers (r=0.81) and intra-interviewers within a 10 to 14 day-interval (r=0.72). Surgery effect size was 1.55. Minimally important difference was 14 points; and scores up to 10 points were considered normal.

**Conclusion:**

The Brazilian Portuguese SNOT-22 version is a valid instrument to assess patients with chronic rhinosinusitis and nasal polyps.

## INTRODUCTION

Quality of life questionnaires have been frequently used in clinical assays in order to determine the impact caused by an intervention or in order to assess healthcare results^1^. The goal of each questionnaire may be geared towards the health status, or quality of life. Health status may be described by physical limitations, functional handicaps or social experiences reported by the patients. Notwithstanding, the quality of life description is seen as a unique and personal experience, which reflects not only the health status, but also other factors and circumstances associated with the patient's life. According to this definition, physicians and other healthcare professionals may describe the health status of each individual, but it is only the patient, individually, who can describe his/her own quality of life^2^.

Chronic rhinosinusitis (CRS), with or without nasal polyps, is a prevalent disease, which can affect up to 14% of the North-American population, and cause a significant impact on quality of life^3^. Such impact may be demonstrated using quality of life questionnaires, such as the SF-36; and patients with CRS have impacts on physical pain and on social aspects which are even greater than patients with angina, congestive heart failure and chronic obstructive pulmonary disease^4^. Moreover, when we compare patients submitted to sinonasal surgery with a sample of the general population in the USA, we notice significant differences in physical pain, general health status, vitality and social aspects^4^.

General questionnaires, such as the SF-36, allow us to compare different situations and treatments, besides enabling us to establish the impact different diseases have in different groups of patients. On the other hand, specific disease questionnaires may identify more easily the important symptoms; they help focus the medical visit and may be used to define treatment objectives. Moreover, they are more sensitive to small changes after interventions than the general questionnaires. Therefore, specific questionnaires are preferrable^5^.

Two recent reviews have identified numerous outcome measure questionnaires used to assess patients with rhinitis and rhinosinusitis, both acute and chronic, evaluating them as to reliability, validity, responsiveness and ease of use^6^,^7^. As more outcome measure questionnaires are developed, there is the need to have an agreement among the many authors in the world, so as to standardize the assessment of rhinosinusitis patients. The SNOT-22 (*Sinonasal Outcome Test*) questionnaire has the advantage of combining issues which are specific of sinonasal disease with general health issues, which may be assessed alone or together, both in the pre and postoperative. Comparing the 15 sinonasal questionnaires, Morley & Sharp^6^ concluded that the SNOT-22 is the most adequate to assess patients with CRS, including the post-op of functional endoscopic surgery.

SNOT-22 is a modification of a pre-existing questionnaire, the SNOT-20, which is a modification of the RSOM-31 (*Rhino-Sinusitis Outcome Measure*) questionnaire of 31 questions. Based on the RSOM-31 validation work, 11 questions were taken out for being considered redundant and of little help; thus making up the SNOT-20. Moreover, the way to calculate the final score was simplified, it is only the summation of the scores from each question; however, with one relevance classification: the patient must point to the five items which are the most important for him/ her. The scores from each question vary between 0 and 5 - meaning that higher scores mean greater problems. The impact of each treatment is measured by the difference in pre and post treatment scores^2^.

In order to create the SNOT-22, the classification of relevance was removed, and two new questions were included (nasal obstruction and reduction of olfaction and taste) because of the concern associated with content validity, i.e., the instrument's capacity to properly measure all the important aspects associated with the disease at hand; bearing in mind that nasal obstruction is the symptom which most forces patients to seek otorhinolaryngological care, and hyposmia is a symptom which does not frequently improve after surgery^3^.

Since this is a questionnaire in English, in order to use it in our country we need to translate it into Brazilian Portuguese. Nonetheless, a simple translation may not be effective, because of cultural and linguistic differences between the peoples. Moreover, the meaning of quality of life and the ways through which health problems are expressed vary between different cultures^1^. For this reason, there is the need to translate and to culturally adapt the SNOT-22 for the Brazilian setting.

The goal of the present paper is to do the translation, cultural adaptation and validation of the SNOT-22 questionnaire from English into Brazilian Portuguese (BR).

## MATERIALS AND METHODS

This study was approved by the Ethics in Research Committee of the institution, under protocol No. 0516/11, and the participants signed an informed consent form.

### Translation

The translation of the quality of life questionnaires required five main stages: (1) translation and (2) retranslation, (3) review by a translation and retranslation committee, (4) equivalence pre-test with bilingual individuals, and (5) re-examination of the scores' weights, when relevant, as proposed by Guillemin^1^.

### Recruitment of subjects

After the translation, we started to recruit the sub-jects. Our sample was made up by patients being followed at the Rhinology Ward of the institution, diagnosed with chronic rhinosinusitis or sinonasal polyps (SNP), submitted to endoscopic sinonasal surgery. As exclusion criteria, we considered age below 18 years and the wish not to participate in the study.

The diagnosis of CRS and SNP was based on the European Consensus on Rhinosinusitis: inflammation of the nose and paranasal sinuses, characterized by two or more symptoms, one of them being nasal obstruction or rhinorrhea, besides facial pain and reduction in the sense of smell, for at least 12 consecutive weeks. The endoscopic exam of the nose may reveal mucous-purulent discharge from the middle meatus or edema and middle meatus block. In SNP, polyps may be present in both middle meatuses.

Eligible patients who consented with the study, answered the SNOT-22 questionnaire prospectively in the preoperative and 3 months after surgery. The total score may vary between 0 and 110, and higher scores mean worse quality of life associated with health. Moreover, in the post-op the patients answered as to the transition classification, i.e., if after the surgery they were: (1) much better, (2) a little better, (3) the same, (4) a little worse or (5) much worse, when compared to before surgery.

The test-retest reliability was carried out in a separate sample of patients with CRS or SNP. The SNOT-22 questionnaire was employed twice during the routine visit of the patient, by different physicians. 10 to 14 days afterwards, the patient answered the questionnaire again, by telephone, to one of the physicians.

The SNOT-22 scores in a normal population, knowingly without sinonasal disease was calculated. The subjects who participated in the study were recruited among members of the medical staff, hospital staff and students from a university, and companions to patients seen in our service. The volunteers were asked whether they suffered from or had suffered rhinitis, chronic rhinosinusitis or sinonasal polyps, and whether they were using nasal medication, and were taken off the study when answered yes to any of these questions.

### Data analysis

The reliability was analyzed in two ways: internal consistency and test and re-test reproducibility. Internal consistency has to do with the way with which each question is associated with the others in the questionnaire, because there has to be homogeneity among the items, which is measured by the Cronbach's alpha coeficient^3^. The minimum acceptable value is 0.7^3^. The test and retest reproducibility measures the stability of an instrument along time after repetitive tests, and it is evaluated by the use of the questionnaire in different occasions, examining the correlation among the scores. The test and retest correlation must be of, at least, 0.7.

The validity of the measures is the capacity the questionnaire has to reflect differences between known groups, using the non-paired T-test. We will analyze the questionnaire's capacity to produce different scores between the group of patients with CRS and SNP, and the group of volunteers without sinonasal disease.

Responsiveness is the questionnaire's capacity to detect clinical changes with time. We will compare the pre and postoperative scores using the paired T-test. Responsiveness can also be assessed measuring the magnitude of the effect, which is the mean value of the scores variation divided by the standard deviation of the initial values. By convention, an effect magnitude between 0.2 and 0.5 is considered a mild improvement; between 0.5 and 0.8, moderate improvement; and greater than 0.8, a great improvement in quality of life^3^.

Clinical interpretability can be calculated by the minimally important difference (MID)^8^, which is the best score difference that a group of patients can detect as real improvement. For that, the patients will be broken down into transition groups, telling whether or not they are, after the procedure: (1) much better, (2) a little better, (3) the same as before, (4) a little worse or (5) much worse. The score variation mean value will be calculated for each transition group. The MID will be the score variation mean value of the “little better” class subtracted from the score variation mean value of the “same as before” class^3^.

For statistical purposes, values of *p*<0.05 were considered significant results.

## RESULTS

### Translation

**Chart 1:**
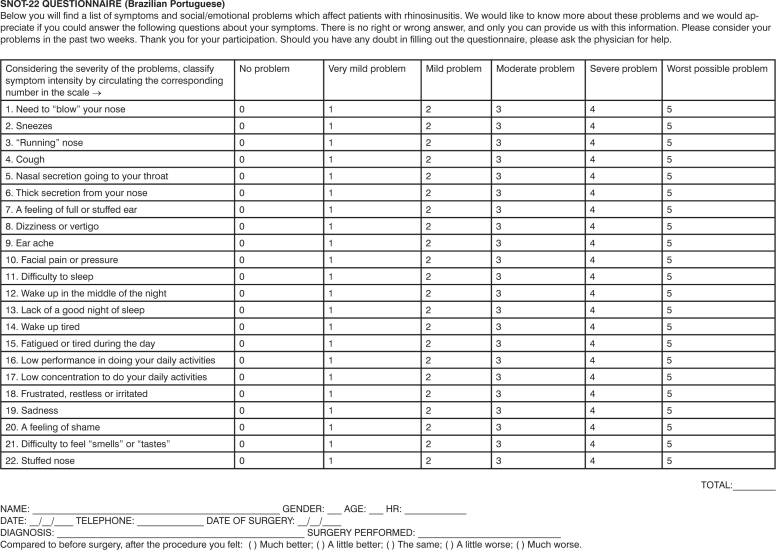
Portuguese Language Version of the SNOT-22.

Chart 1 shows the results from the SNOT-22 translation and cultural adaptation.

### Patients' characteristics

We grouped 89 patients who were submitted to sinonasal functional endoscopic surgery and filled out the questionnaire before and after surgery; 56.18% were women, with mean age of 44.87 years (18 to 70; SD=13.52). Of the 89 patients, 44 (40.44%) had SNP, with mean age of 49 years (18 to 70; SD=11.36) and 45 (50.56%) had CRS, with mean age of 40.65 years (18 to 68; SD=14.35).

Among volunteers without sinonasal disease, we recruited 113 people, 56.64% women, with mean age of 23.35 years (18 to 61; SD=8.13).

### Data obtained

The internal consistency of the questionnaire in the preoperative period was high, with a Cronbach's alpha coefficient of 0.9276. As we measure the internal consistency, removing one question at a time, we noticed alpha values varying between 0.9198 and 0.9301, showing that all questions were homogeneous.

The test-retest reproducibility was assessed with 24 CRS or SNP patients. The questionnaire was deployed by two different physicians, during consultation, resulting in a correlation of 0.81. The questionnaire use by one of the two physicians by phone, after 10 to 14 days had a correlation coefficient of 0.72.

The mean values of the total scores from the groups studied (without sinonasal disease, in the pre-op and post-op) are presented on Table 1. The instrument was capable to differentiate the groups studied, demonstrating its validity (*p*<0.0001 for each comparison). Even considering the SNP and CRS groups separately in the pre and post-op, there was a difference between these groups and the group without sinonasal disease (Tables 2 and 3).

**Table 1:** Characteristics of the total scores of the groups studied.

Group	N	Mean	Median	SD	CI 95%
Without sinonasal disease	113	11.42	10	9.46	11.37 to 11.48
Pre-op CRS + SNP	89	62.39	68	25.30	62.23 to 62.56
Post-op CS + SNP	89	23.09	17	18.79	22.96 to 23.21

T-Test: WITHOUT *vs*. PRE *p*<0.0001; WITHOUT *vs*. POST *p*<0.0001

CRS = Chronic Rhinosinusitis

SNP = Sinonasal polyps

N = Number

SD = Standard deviation

CI 95% = Confidence Interval of 95%

**Table 2:** Comparison of pre-op scores.

Group	N	Mean	Median	SD	CI 95%
Without sinonasal disease	113	11.42	10	9.46	11.37 to 11.48
SNP preoperative	44	60.18	63.5	27.52	59.92 to 60.44
CRS preoperative	45	64.56	68	23.02	64.34 to 64.77

T-Test: WITHOUT *vs*. PRE *p*<0.0001; WITHOUT *vs*. POST *p*<0.0001

CRS = Chronic Rhinosinusitis

SNP = Sinonasal polyps

N = Number

SD = Standard deviation

CI 95% = Confidence Interval of 95%

**Table 3:** Comparison of postoperative scores.

Group	N	Mean	Median	SD	CI 95%
Without sinonasal disease	113	11.42	10	9.46	11.37 to 11.48
Post-op SNP	44	16.02	13	12.02	15.91 to 16.14
Post-op CRS	45	30.00	28	21.60	29.80 to 30.20

T-Test: WITHOUT *vs*. POST SNP *p*=0.02; WITHOUT *vs*. POST CRS *p*<0.0001

CRS = Chronic Rhinosinusitis

SNP = Sinonasal polyps

N = Number

SD = Standard deviation

CI 95% = Confidence Interval of 95%

The mean value of the scores from each question in each of the groups studied is presented on Table 4, which also shows the mean variation between pre and post-op scores for each question.

**Table 4:** Mean scores by question per group studied.

	SNOT-22 Mean Scores			
Question	Without Disease	Pre-op	Post-op	Difference Pre-Post
1.	0.62	3.00	1.43	1.57
2.	0.66	3.00	1.69	1.31
3.	0.50	3.21	1.10	2.11
4.	0.60	1.79	1.12	0.66
5.	0.47	3.31	1.37	1.94
6.	0.22	2.76	1.33	1.44
7.	0.39	2.66	0.98	1.69
8.	0.29	1.48	0.79	0.70
9.	0.19	1.43	0.65	0.78
10.	0.12	2.81	0.99	1.82
11.	0.43	3.70	0.96	2.74
12.	0.30	3.47	1.25	2.22
13.	0.74	3.19	1.01	2.18
14.	0.96	2.73	0.88	1.85
15.	1.09	2.64	1.00	1.64
16.	0.78	2.75	0.82	1.93
17.	0.92	2.35	0.97	1.38
18.	0.66	3.42	0.90	2.52
19.	0.37	2.55	0.62	1.93
20.	0.19	2.22	0.27	1.96
21.	0.36	3.66	1.69	1.98
22.	0.53	4.25	1.30	2.94

Comparison by question:

Pre vs. Post: *p*<0,0001 in all the questions

Pre vs. Without : *p*<0,0001 in all the questions

Post vs. Without: *p*<0,0001, except for questions 13 to 20, not significant

The statistically significant reduction in the postoperative scores, vis-à-vis preoperative values, demonstrates the instrument's responsiveness, as we can see on Table 5. The magnitude of the surgery's effect after 3 months was 1.55 - considered high (>0.8). In patients with CRS, the magnitude effect was 1.50 and in those with SNP, the effect was higher: 1.60.

**Table 5:** Responsiveness.

	SNP			CRS			SNP + CRS		
Measure	N	Mean	SD	N	Mean	SD	N	Mean	SD
Pre-op	44	60.18	27.52	45	64.56	23.02	93	62.39	25.30
Post-op	44	16.02	12.02	45	30.00	21.60	93	23.09	18.79
Difference Pre-Post	44	44.16	23.73	45	34.56	21.03	93	39.30	22.80

T-test between PRE *vs*. POST: SNP *p*<0.0001; CRS *p*<0.0001; SNP+CRS *p*<0.0001

CRS = Chronic Rhinosinusitis

SNP = Sinonasal polyps

N = Number

SD = Standard deviation

We obtained the classification as to the clinical condition after surgery from 81 patients. The mean score variation in the pre and post-op from each transition group can be seen on Table 6. The minimally important difference was 13.87 points. This means that changes smaller than 14 points in the SNOT-22 may not be seen as improvement or worsening for the patient.

**Table 6:** Difference in the pre and post scores per transition group.

Transition groups	N	Pre-Post Difference	SD
Much better	48	42,94	25,21
A little better	10	21,70	21,78
The same	12	7,83	14,76
A little worse	5	-1,40	1,95
Much worse	6	-16,17	9,43

N = Number

SD = Standard deviation

## DISCUSSION

Outcome questionnaires are usually employed by self-administration, as the origin questionnaire of the present study^3^. Nonetheless, given the difficulties in reading and understanding the text by part of the population seen in our service, we decided to standardize the way through which the SNOT-22 questionnaires are administered: we read the questions to the patients, as it has been done by some Brazilian authors^9^,^10^. The administration of the questionnaire to the patients by the examiner has some advantages over self-administration, such as faster filling out time, lower rate of missing data, and interviewees' preference^11^. Moreover, we believe that this way of using the questionnaires can reduce possible differences in results which its administration by telephone (in the test-retest reproducibility evaluation) could bring about, so much so that the questionnaire demonstrated a high reproducibility rate. Despite difficulties presented by some patients, nonspecific questions presented heterogeneity in relation to the others, with a high Cronbach's alpha coefficient.

Responsiveness is one of the main qualities of the SNOT-22. Its predecessor - SNOT-20, had an important drawback because it is not very sensitive to clinical changes^2^,^.3^,^5^. The introduction of questions about hyposmia (question 21) and nasal obstruction (question 22) have clearly increased the questionnaire's validity, because they are two very important complaints that motivate the patient to seek medical care^3^. Our study has shown that the Brazilian version of the SNOT-22 is efficient to measure changes after surgical treatment. Question 22 was the one showing the greatest difference between pre-op and postop situations (2.94 points), demonstrating its importance in this questionnaire. The mean score reduction was 39.53 points after surgery, with a greater impact on patients with SNP (a reduction of 44.16 points). The original SNOT-22, in English, demonstrated a lower reduction in the postoperative, but with a similar pattern (greater reduction in patients with SNP)^3^. The effect magnitude was considered high in our study as well as in the original study with the SNOT-22 in English (1.57 and 0.81, respectively), also with a higher effect on patients with SNP (1.60 and 0.90, respectively)^3^.

The SNOT-22 proved capable of differentiating groups of patients with sinonasal diseases from individuals without nasal disease. The mean values of the scores from patients with CRS or SNP are far from the mean score of healthy individuals. Our sample of healthy individuals had a mean score of 11.42 points, close to the one presented by Hopkins et al.^3^ - 9.3. A study from the same group advocated the use of the median as normality threshold, instead of the mean, since SNOT-22 values in healthy patients do not tend to normal distribution. Therefore, they considered 7 as the normality threshold for the SNOT-22^12^. Utilizing such criterion, the normality threshold for the Brazilian SNOT-22, given our sample, would be 10. With that, we would have only two patients with normal SNOT-22 indices in the pre-op and 24 in the post-op, of the 93 operated individuals. Even after surgery, the mean scores had significant differences between sick and healthy individuals, demonstrating how the instrument can capture differences between these groups, including after surgery. We chose to use a group without sinonasal diseases with a lower mean age than the study group, because since the questionnaire involves general and specific questions, a younger group would tend to present lower scores, they would present lower general complaints.

Clinical interpretability is the main challenge for the researchers interested in measuring life-quality ques-tionnaires, because these questionnaires do not produce intuitively significant data, making it difficult to interpret the clinical importance of the differences between groups and individuals. Our study estimated the minimally important difference to be 14 points, near the estimated value in the original SNOT-22 in English, which was of 9 points^3^. This means that, in our questionnaire, variations up to 14 points may not be interpreted as improvement or worsening by the patient.

## CONCLUSION

The Brazilian Portuguese version of the SNOT-22 is a valid instrument to assess patients with CRS and SNP, it demonstrated internal consistency, reproducibility, validity and responsiveness.
